# Clinical application of Kirschner wires combined with 5-Ethibond fixation for patella fractures

**DOI:** 10.3389/fsurg.2022.968535

**Published:** 2023-01-06

**Authors:** Yuan Liang, Jinlong Hu, Pei Zhang, Jiale Zhang, Lixun Yang, Wendong Zhang, Jiaxin Chen, Jinshan He, Yongchao Fang, Yuelai Zhou, Pengtao Chen, Jingcheng Wang

**Affiliations:** Department of Orthopedics, Clinical Medical College of Yangzhou University, Northern Jiangsu People's Hospital, Yangzhou, China

**Keywords:** patella fracture, suture, tension band, Kirschner wire, 5-Ethibond

## Abstract

**Background:**

Patella fractures that require surgery are conventionally treated using Kirschner wires (K-wires) and stainless steel wires. In recent years, the nonabsorbable polyester has been reported to have excellent outcomes clinically. Therefore, the goal of our study was to evaluate the effects of Kirschner wires combined with 5-Ethibond on treating patellar fractures.

**Methods:**

From July 2018 to January 2022, 22 patella fracture patients were treated with Kirschner wires combined with 5-Ethibond. Radiographs of the knees were used to evaluate fracture healing and hardware complications. The clinical results were evaluated through the functional score, knee joint range of motion (ROM), and Bostman patella fracture functional score.

**Results:**

The average age of patients was 57.4 ± 11.9 (range 33–74) years. The mean follow-up time was 15.2 ± 7.6 (range 4–36) months. The mean operation time was 56.8 ± 8.7 (range 45–80) min. The entire patients had bony union at an average of 10.5 ± 1.9 (range 8–14) weeks. At the final follow-up, the mean range of postoperative ROM was 123.4° ± 14.6° (range 95°–140°), and the functional score was 28.7 ± 1.2 (range 26–30) points. No patient exhibited internal fixation failure, and no symptomatic implants or skin complications were recorded.

**Conclusions:**

The fixation approach using K-wires combined with 5-Ethibond has a lower complication rate and delivers superior clinical results. This research reveals that such technology is a safe and prospective substitute for conventional metal fixation approaches.

## Introduction

Patella fractures account for approximately 1% of all skeletal fractures (1, 2) and are usually caused by the forceful contraction of the quadriceps femoris. These fracture features can be transverse, perpendicular, or stellate. Open reduction and internal fixation (ORIF) is necessary for fractures that have fragment displacement >3 mm, joint incongruence >2 mm, or a disrupted extensor mechanism. The aims of surgical treatment are to achieve the stable fixation of fragments, anatomical reduction of the articular surface, and early restoration of range of motion (ROM) (3, 4).

There are numerous methods for the internal fixation of patella fractures, and tension band wiring (TBW) technology is the most extensively utilized approach. Such technology contains longitudinal Kirschner wires (K-wires) inserted across the fracture and a stainless steel wires (SSW) looped in an eight-like fashion over the anterior patella. Nevertheless, various SSW-related complications have been reported, including wire fatigue failure, wire irritation, and delayed wound healing (5, 6).

Consequently, several modifications have been introduced to avoid steel wire-related complications by using strong suture materials along with other tension band suture techniques (7).

At our department, our team used a modified tension band technology for treating patella fractures with K-wires combined with 5-Ethibond. This retrospective research is intended to clinically investigate the radiographic outcomes of K-wires combined with 5-Ethibond in the therapy of patella fractures.

## Materials and methods

### Patient characteristics

This research was approved by the Ethical Committee of the Clinical Medical College of Yangzhou University, and every patient offered informed consent. We collected retrospective data from all patients with displaced intra-articular patella fractures between July 2018 and January 2022. Preoperative x-ray and three-dimensional computed tomography (CT) scanning images were acquired to assess patella fractures. The inclusive criteria were as follows: all patients were older than 18 years; the individual with an acute closed patella fracture featuring an articular incongruity >2 mm or a separation of fragment fracture >3 mm; the general physical condition of the patient could withstand the anesthesia and operation; and fractures without any deep infection. The exclusion criteria were individuals who were younger than 18 years, simple or nondisplaced fractures with indications for conservative treatments; patients with open fractures, periprosthetic fractures, and pathologic fractures; patients who were followed up for less than 3 months; the general physical condition of the patients could not withstand the anesthesia and operation; patients receiving revision procedures; patients with knee function limitation or other severe medical conditions before injury; and individuals with multiple concomitant injury of the ipsilateral leg or other systems. Patients were treated with TBW with K-wires and 5-Ethibond. Patients' demographic information and operational information were obtained and analyzed based on the database of our hospital.

### Surgical procedures

**Figure 1:**
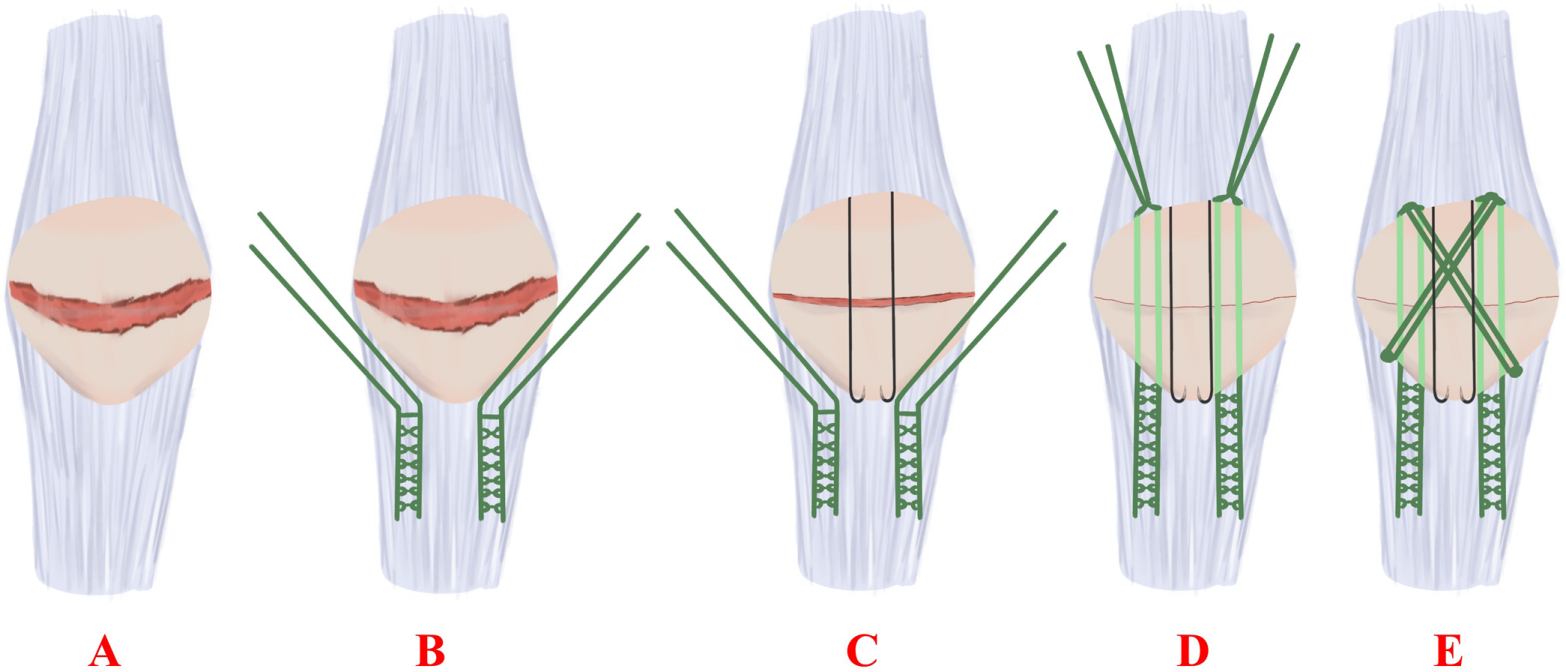
(**A–E**) Illustrations: surgical technique.

Every patient was treated with a single dosage of cefazolin (50 mg/kg, a maximal dosage of 2 g) antibiotic for antimicrobial prophylaxis within the 30-min incision. After anesthesia, the patient was put on the operation table in the dorsal position. Then, a longitudinal midline incision was made over the knee to expose the fractured site. Following that, the intra-articular hematoma and blood clots were cleaned; afterwards, a 5-Ethibond was introduced in a Krackow manner up and down the medial and lateral edges of the patellar tendon, during which two sharp reduction clamps were used to temporarily maintain reduction. The two or three 1.6-mm K-wires (Youke, Shanghai, China) were introduced in parallel by inserting them from the upper pole to the lower pole of the patella. The remaining four suture limbs passed through the patella. The sutures were afterwards tied at the superior pole with the knee in extension. The remaining 5-Ethibond was then passed through the front of the patella to form an eight-like shape (Figure 1); then, the retinaculum was repaired. During the surgery, the active knee joint displayed stable fracture, and x-ray fluoroscopic results demonstrated an excellent decrease in the fracture.

### Postoperative management and evaluation

Postoperatively, x-ray examinations were completed the day after surgery. Generally, flexion in-brace (0°–90°) was allowed for 0–2 weeks postoperatively. Patients were regularly followed up every 4–6 weeks. Before the patient discharged from hospital, we told the patient that the rehabilitation plan was as follows: the knee ROM was progressively elevated, and patients exercised themselves with the objectives of realizing 90° flexion by the 28th day, 120° flexion by the 42nd day, and full ROM eventually. The demographic data of patients includes age, sex, trauma mechanisms, fracture types, and length of follow-up. Follow-up records includes fracture union time, knee joint ROM, Bostman score, and complications.

Radiographic images were analyzed to evaluate the original fracture types, displacements, implant positions, and treatment time.

### Statistics

All data were described as the averages ± *SD*, and our statistic assay was completed *via* SPSS 26.0.

## Results

The 22 patients were enrolled in the present research (7 males and 15 females). The average age of the patients was 57.4 ± 11.9 (range 33–74) years. The mean operation time was 56.8 ± 8.7 (range 45–80) min. The mean follow-up time was 15.2 ± 7.6 (range 4–36) months. The demographic information is further demonstrated in Table 1.

**Table 1:** Patients demographic.

Case No.	Age	Sex	Mechanism of injury	Fracture type	Operation time (min)	Follow-up (months)
1	64	Female	Fall	Comminuted displaced	60	22
2	40	Male	Fall	Comminuted displaced	80	12
3	51	Female	Fall	Transverse with inferior pole comminution	70	10
4	41	Male	Fall	Simple transverse	50	13
5	73	Female	Fall	Comminuted displaced	60	5
6	50	Male	Fall	Comminuted displaced	55	18
7	66	Female	Fall	Simple transverse	50	23
8	52	Female	Fall	Simple transverse	55	26
9	57	Female	Fall	Transverse with inferior pole comminution	60	18
10	51	Male	Fall	Transverse lower 1/3	55	14
11	73	Female	Fall	Comminuted displaced	60	18
12	62	Female	Fall	Simple transverse	50	15
13	57	Female	Fall	Simple transverse	45	7
14	33	Male	Fall	Transverse lower one-third	45	15
15	38	Female	Fall	Comminuted displaced	60	12
16	53	Female	Fall	Comminuted displaced	70	36
17	64	Female	Fall	Comminuted displaced	45	18
18	65	Male	Fall	Comminuted displaced	55	17
19	64	Female	Fall	Comminuted displaced	60	10
20	68	Male	Fall	Comminuted displaced	55	18
21	66	Female	Fall	Simple transverse	50	4
22	74	Female	Fall	Comminuted displaced	60	4

**Figure 2:**
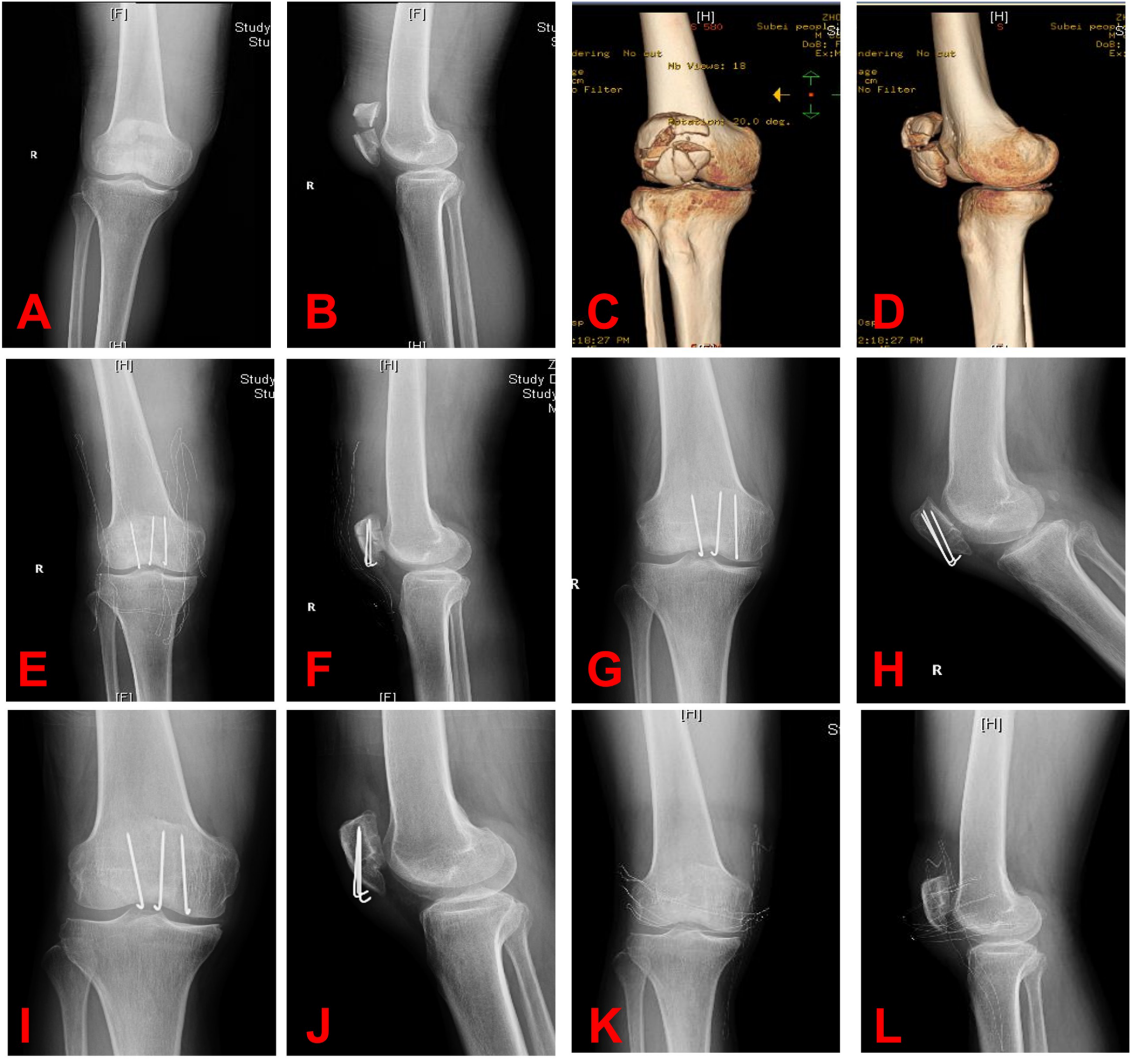
(**A–D**) Radiographs of comminuted patella fracture. (**E,F**) In this comminuted patella fracture, we used the 5-Ethibond and three K-wires. (**G,H**) After 3 months, the radiograph revealed a good union of fracture. (**I,J**) After 12 months, no breakage, loosening, or migration of the internal fixation was detected, and a good union of fracture was seen. (**K,L**) Radiograph after implant removal.

**Figure 3:**
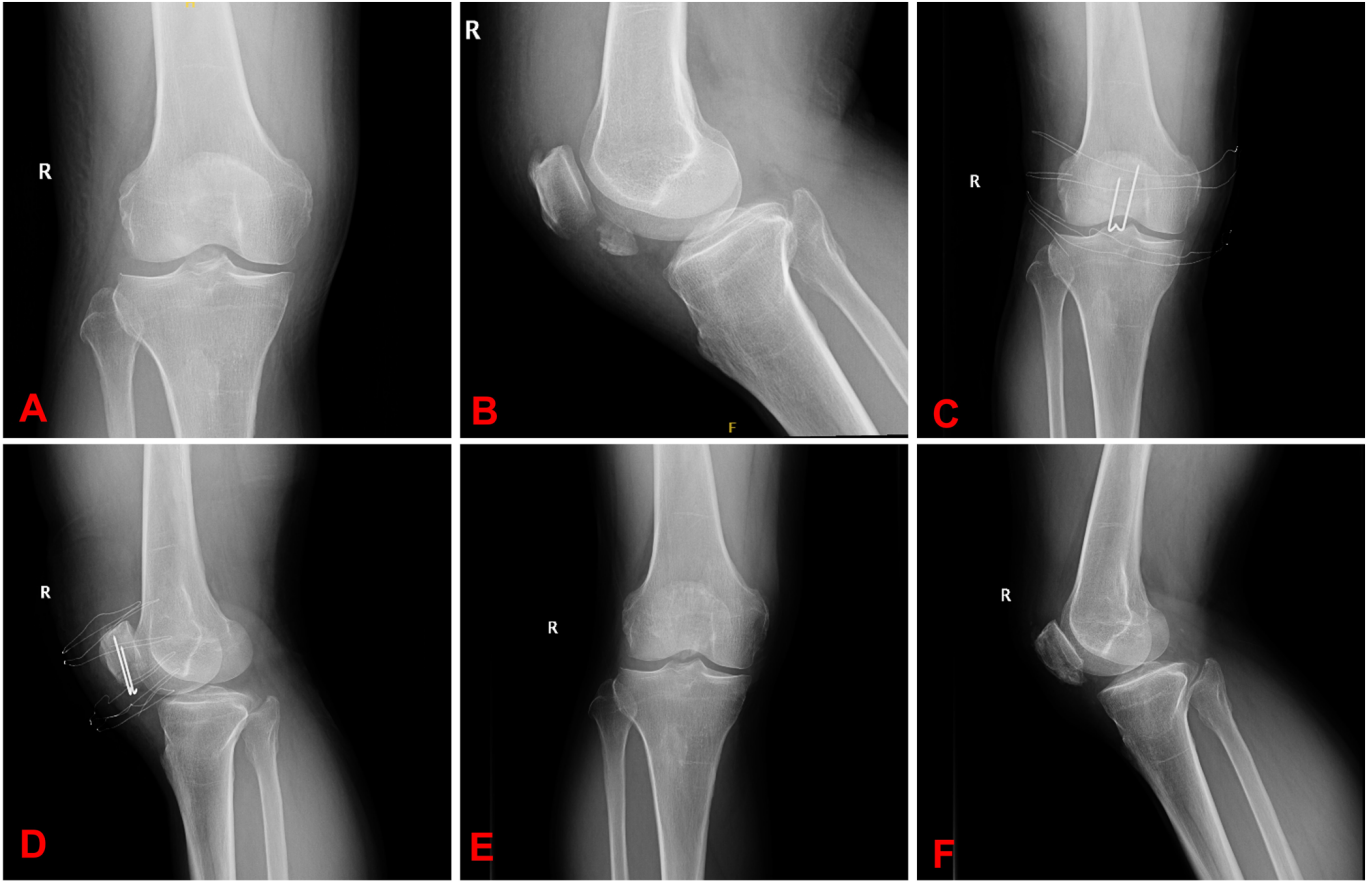
(**A,B**) Radiographs of distal patellar fracture. (**C,D**) In this distal patella fracture, we used the 5-Ethibond and three K-wires. (**E,F**) Radiograph after implant removal.

**Figure 4:**
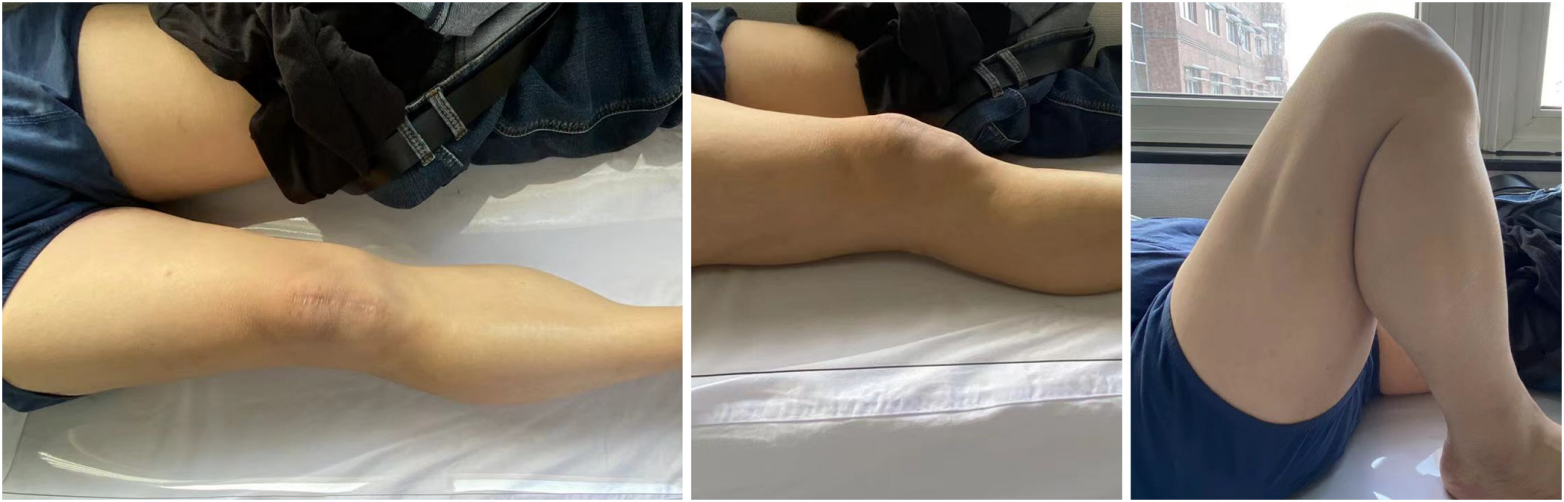
Final follow-up clinical photographs showing satisfactory extension and flexion.

All patients had bony union at an average of 10.5 ± 1.9 (range 8–14) weeks. There was no loss of reduction during follow-up. No breakage, loosening, migration, and skin irritation of the inner fixation were observed during the follow-up (Figures 2, 3). At the eventual follow-up, the mean postoperative ROM was 123.4° ± 14.6° (range 95°–140°) (Figure 4); we have not observe the K-wire slippage in all cases so far. The Bostman patella fracture function score was 28.7 ± 1.2 (range 26–30). The clinical outcomes of patients are displayed in Table 2.

**Table 2:** The clinical outcomes.

No. of patients	22
Fracture union time (weeks)	10.5 ± 1.9
Final ROM (°)	123.4 ± 14.6
Functional score	28.7 ± 1.2
Complications	No

## Discussion

The main aim of operative treatment is to reduce the anatomic articular surface, achieve stable patella fixation, and facilitate early knee mobilization (8). The TBW technology is the most extensively utilized surgical fixation approach to stabilize patella fractures. Traditional tension band construction uses two K-wires and a metal low-gauge wire tension band. After fracture reduction, the K-wires were introduced into the fracture fragments along the subchondral surface *via* insertion. Then, the stainless steel wire was placed in an eight-like fashion to realize fracture compression. Although stainless steel wires can provide stable fixation, these wires are usually associated with hardware-induced symptoms and other complications (9–11). Meena et al. discovered three probable factors that might break wires: (1) repeated stress on the wires because of a remarkable ROM of knee joints; (2) utilization of thin wires; and (3) utilization of low-quality hardware (12). As a result, it is imperative to explore sutures in the fixation techniques instead of investigating stainless steel wires.

Using sutures can deliver several advantages compared with stainless steel wires (9, 13). Harrell et al. concluded that several loops of Ethibond could replace SSWs, but such a method might not be good for the tension band fixation of fractures (14). Patel et al. compared 5-Ethibond with SSWs in the treatment of patella fractures *via* the adjusted tension band technology and the Lotke technology in a biomechanical study. The result revealed that the 5-Ethibond was able to withstand loads comparable to those of SSWs for patella fractures (15).

In 2001, Gosal et al. compared patellar fracture fixation based on metal wires or 5-Ethibond. They recorded a 38% hardware removal rate due to pain and hardware failures when metallic implants were utilized, and they did not record any symptoms associated with the utilization of heavy sutures. The suture fixation group, nevertheless, displayed a 6% failure rate, which indicated that the substance and approach utilized could induce fixation failures prior to the use of traditional metal implants (16). This is probably the reason why sutures are not usually suggested for treating patellar fractures. We proposed that the failures of suture fixation were due to the lack of metallic parallel longitudinal intraosseous fixation.

Hence, instead of stainless steel wires, we selected K-wires combined with 5-Ethibond to treat patella fractures in this study.

Lee et al. reported that using the adjusted tension band technology with FiberWire and retaining metal parallel implants like K-wires could achieve good results (17). No patient had broken wires, and nonunion with deformity was observed in one individual. Bryant et al. contrasted fracture fixation technologies with the anterior tension band through cannulated screws by virtue of FiberWire and SSWs. The research outcomes presented no remarkable diversity between the FiberWire group and the SSW group (18).

In our study, to obtain a better stability of fracture fixation, the remaining suture limbs were passed through the patella. Then, sutures were tied at the superior pole with the knee in extension. The remaining 5-Ethibond was then passed through the front of the patella to form a certain shape. The most important discovery of our study was that using K-wires combined with 5-Ethibond was a valid, secure, and easy therapeutic method for patella fractures. The average operation time was 56.8 minutes, which indicated that such a surgical approach might not bring additional technological challenges or tissular injuries. Hence, this method is quite prospective and can be utilized extensively. In the present research, patients received recovery training and full weight bearing training as early as the second day after surgery. Clinically and radiologically, the outcomes revealed that such technology could deliver satisfactory results for patients. No patient experienced the loss of reduction and inner fixation failures, and no patient received a remedial operation. Those outcomes coincide with previously completed research that revealed that nonabsorbable sutures could substitute for metallic wires due to their strength. Hence, the 5-Ethibond can offer similar protective effects as steel wires in antagonizing displacements and improving strength in knee extensor loading.

We believe that the adjusted tension band techniques based on 5-Ethibond are simple and can decrease irritation and the quantity of broken wires. Moreover, these methods may be more valid in contrast to the single use of nonabsorbable polyester due to the probability of early recovery and better life quality.

Nevertheless, there are certain flaws in the present research. First, our study was finished retrospectively. Second, the number patients was insufficient. Third, our team did not complete the biomechanical analysis of our construct to evaluate its biomechanical strength. Fourth, during the research, many patients, even asymptomatic patients, wished to remove implants owing to culture-related reasons. Hence, more high-quality, double-blinded randomized clinical trials with larger sample sizes are warranted to substantiate the benefits of fixation using K-wires combined with 5-Ethibond.

## Conclusions

Patella fracture patients treated with K-wires combined with 5-Ethibond presented favorable clinical outcomes. This technique may be a valid fixation approach for healing displaced and comminuted patella fractures.

## Data Availability

The original contributions presented in the study are included in the article/Supplementary Material, further inquiries can be directed to the corresponding authors.
